# Idiopathic Retroperitoneal Fibrosis Presented As Urinary Tract Obstruction

**DOI:** 10.7759/cureus.29582

**Published:** 2022-09-26

**Authors:** Darshankumar M Raval, Vaishnavi M Rathod, Milauni Dave, Nilay S Patel, Riya Dobariya

**Affiliations:** 1 Department of General Medicine, Sir Sayajirao Genera (SSG) Hospital, Medical College Baroda, Vadodara, IND

**Keywords:** hydroureteronephrosis, urinary tract obstruction, ormond's disease, acute kidney injury, idiopathic retroperitoneal fibrosis

## Abstract

Retroperitoneal fibrosis (RPF) or Ormond’s disease is a very uncommon fibro-inflammatory disease, under the umbrella of systemic autoimmune diseases. The majority of cases are idiopathic, known as idiopathic RPF (IRPF); however, diseases secondary to other causes are also seen in clinical practice. The commonest presenting features are seen due to the effects of fibrous tissue around iliac vessels, aorta and ureters, where compression of ureters is the major and most common complication. Computed tomography (CT) scans and magnetic resonance imaging (MRI) are the modalities of choice for the diagnosis. The primary management involves medical therapy with corticosteroids and reserving surgical options for ureteric obstruction and related complications. We present a case of a 65-year-old man who presented with bilateral pedal oedema, facial puffiness, decreased appetite, decreased urine output, and breathlessness with dry cough, tachypnoea, hypoxia and crepitation in both lung fields on examination. The blood investigations were suggestive of acute kidney injury (AKI); whereas radio imaging diagnosed him as a case of bilateral hydroureteronephrosis with RPF. The patient was treated for AKI in the case of IRPF. Once the patient stabilized, a low-dose systemic steroid was started for IRPF, and subsequently, the patient underwent stent placement surgery for ureteric obstruction. RPF, being a rare disease, is difficult to diagnose. However, CT and MRI scanning can easily reveal fibrous tissue surrounding the aorta and ureters. Medical management with glucocorticoids is the backbone drug for the disease, keeping surgery as a reserved option for ureteric obstruction and its complications.

## Introduction

Retroperitoneal fibrosis (RPF), also known as Ormond’s disease, is an uncommon fibro-inflammatory disease of which pathogenesis is still unclear, involving the retroperitoneal tissue surrounding the infrarenal aorta, specifically around the lumbar region (L4- L5 vertebrae), notably obstructing ureters. Under the spectrum of systemic autoimmune diseases, its cause is idiopathic in about two-thirds of cases (70%) (idiopathic RPF, IRPF) with atherosclerosis being the most common predisposing factor. The remaining one-third of cases occur secondary to several factors, such as infections, malignancies, surgery, injuries, occupational exposure (like asbestos), and drugs. For therapeutic purposes, it is important to distinguish between these two forms, as secondary diseases are usually treated by removing an underlying causative factor [1,2].

RPF is most commonly seen in middle-aged patients between 40 and 60 years of age, with male predominance, having a male-to-female ratio of approximately 2:1 or 3:1. The real incidence is not known but is estimated to be one per 200,000 to 500,000 per year [3].

The clinical picture is not specific, the patient presents with a range of symptoms from abdominal pain to symptoms due to ureteral compression, later being the major complication. Imaging modalities such as computed tomography (CT) and magnetic resonance imaging (MRI) are the most reliable test, therefore they are crucial in making the diagnosis. Stopping the fibro-inflammatory reaction to progress further is the goal of the treatment, for which medical management is the first line of treatment, whereas surgery is usually done to remove a ureteral obstruction and should always be given together with systemic steroid therapy [4]. Here, we put forth a case of RPF in a 65-year-old male who presented to us with symptoms and signs of acute kidney injury (AKI) secondary to urinary tract obstruction caused by IRPF.

## Case presentation

A 65-year-old male patient, with no significant past medical or family history, presented to our hospital with complaints of weakness and decreased appetite for the last two months, increased urinary frequency and facial puffiness (predominantly in the peri-orbital region) for one-month, bilateral pedal oedema and dry cough since last 20 days, breathlessness (New York Heart Association- NYHA Grade-3) and decreased urine output since last 4-5 days. There were no complaints of abdominal distension, chest pain, nausea, vomiting, diarrhoea, burning micturition, fever, altered sleep, altered behaviour or convulsion. The patient was a chronic bidi smoker (10-12 bidis/day) and a chronic alcoholic for 40 years.

**Figure 1:**
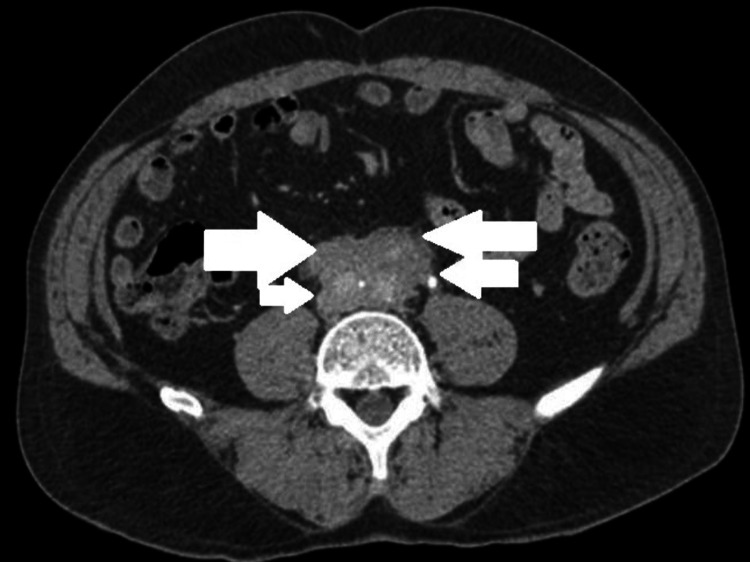
MDCT abdomen and pelvis showing an enhancing soft tissue density mass around abdominal aorta and bilateral common iliac arteries suggestive of idiopathic retroperitoneal fibrosis (IRPF). MDCT - Multi-Detector Computed Tomography

On general examination, the patient had mild tachypnoea and hypoxia, with a respiratory rate of 22 breaths/min and SpO2 of 85% on room air, for which nasal oxygen was started at 4 litre/min. The rest of the general examination was unrevealing except for the presence of bilateral pitting type of pedal oedema up to the knee joint. Thorough systemic examination showed the presence of extensive crepitation in both lung fields with the occasional presence of rhonchi, without significant involvement of other systems. The routine investigations were suggestive of AKI with increased urea, creatinine, phosphorus, uric acid, and decreased ionized calcium (Table 1). When ultrasonography of the abdomen and pelvis was done to look for the kidneys, it showed bilateral hydroureteronephrosis with soft tissue mass surrounding the aorta and bilateral iliac vessels - the possibility of fibrosis. This was further confirmed by CT scanning of the abdomen and pelvis (Table 2, Figure 1).

**Table 1:** Serial routine blood investigations N/L/E/M - Neutrophil/Lymphocyte/Eosinophil/Monocyte, SGPT - Serum glutamic pyruvic transaminase, SGOT - Serum glutamic-oxaloacetic transaminase, I.N.R. - International normalized ratio, HIV - Human immunodeficiency virus, HbsAg - Hepatitis B surface antigen, HCV - Hepatitis C virus

Investigations	On Admission	Day 3	Day 5	Day 7	Day 10
Hemoglobin (gm %)	9.5	10.0	9.3	9.8	10.4
Total Count (per cumm)	7,200	6,900	6,000	7,600	8,100
Differential Count (N/L/E/M %)	78/20/01/01	85/13/01/01	66/32/01/01	76/22/01/01	74/24/01/01
Platelet Count (per cumm)	2,30,000	2,33,000	2,30,000	3,21,000	3,33,000
Urea (mg/dL)	114	111	87	56	32
Creatinine (mg/dL)	3.74	3.55	3.45	2.11	1.89
Bilirubin (mg/dL) Total/ Direct/ Indirect	0.7/ 0.3/ 0.4	0.7/ 0.3/ 0.4	0.6/ 0.2/ 0.4	0.8/ 0.3/ 0.5	0.8/ 0.2/ 0.6
Sodium (mmol/L)	136	131	138	136	134
Potassium (mmol/L)	4.7	3.6	5.4	4.9	3.8
SGPT (U/L)	13				
SGOT (U/L)	56				
Alkaline Phosphate (IU/L)	493				
Total Protein (gm/dL)	5.1				
Albumin (gm/dL)	3.0				
Ionized Calcium (mmol/L)	1.07				
Phosphorus (mg/dL)	11.0				
Uric Acid (mg/dL)	9.9				
Random Blood Sugar (mg/dL)	103				
Prothombin Time (Seconds) Test/ Control	15.10/ 12.20				
I.N.R.	1.24				
Partial Thromboplastin Time (Seconds) Test/ Control	33.10/ 32				
HIV Antibody Test (HIV-1 & HIV-2)	Negative				
HbsAg	Negative				
HCV	Negative				

**Table 2:** Radiological investigations

Investigations	Reports
Chest X-Ray (PA)	Blunting of Left Costophrenic (CP) angle – Pleural Effusion
Ultrasound of Abdomen and Pelvis	Right kidney – Gross Hydronephrosis with Midpole parenchymal thickness of 5 mm and upper hydroureter of 7 mm without evidence of (e/o) calculi. Left kidney – Moderate hydronephrosis with Midpole parenchymal thickness of 10 mm and upper hydroureter of 5 mm without e/o calculi. Bladder – partially distended with wall thickness of 8 mm – possibility of (p/o) Cystitis Enlarged Prostate (46 cc) Bilateral mild to moderate pleural effusion? Soft tissue lesion noted surrounding ureters and aorta - ? Retroperitoneal Fibrosis
2-Dimentional (2D) Echocardiography	No Regional Wall Motion Abnormality. Left Ventricular Ejection Fraction ( LVEF) – 55% - 60%. Normal Left Ventricle (LV) size with Fair LV systolic function. Mild Mitral Regurgitation (MR) . Mild Tricuspid Regurgitation (TR)with Mild Pulmonary Arterial Hypertension (PAH) Grade I LV Diastolic Dysfunction.
Multi-Detector Computed Tomography (MDCT) Abdomen & Pelvis	Enhancing soft tissue density mass (approx. 8.6 cm in length) around abdominal aorta and bilateral common iliac arteries seen to encase the bilateral upper ureter with resultant proximal right gross and moderate left hydroureteronephrosis, suggest p/o Idiopathic Retroperitoneal Fibrosis. Changes of cystitis. Prostatomegaly. Bilateral moderate pleural effusion.

The patient's C-reactive protein (CRP) was 24 mg/L and the erythrocyte sedimentation rate (ESR) was 40 mm after an hour. Considering the diagnosis of IRPF other investigations were done which were suggestive of negative antinuclear antibodies (ANA) profile, normal thyroid function test, and normal immunoglobulin G4 (IgG4) levels (0.99 g/L; normal range: 0.052-1.250 g/L). After history, examination and investigations, the patient was diagnosed with a case of AKI complicated by uremic lungs in the case of IRPF. For bilateral hydroureteronephrosis, urosurgical reference was sought, advising for stent placement in both ureters. The patient was treated for AKI, and a low-dose systemic steroid was started for IRPF once the patient improved clinically. The patient subsequently underwent bilateral ureteric stent placement surgery for a ureteric obstruction within a week of diagnosis of IRPF. Using a cystoscope, double lumen J (DJ) stents were placed in both ureters under general anaesthesia. The whole procedure of stent placement was uneventful without developing any subsequent complications. The position of the stents was confirmed by ultrasonography. The urologist advised keeping the stents in situ till the RPF responds to treatment, therefore relieving obstruction to ureteric flow. After 10 days of hospitalization, the patient’s general condition and blood parameters improved, and the patient was discharged with advice to follow up on monthly basis for renal function monitoring and assessment of response to steroids. As per advice from the urologist, stents were kept in situ till the RPF decreases in size and obstructions on the ureters relieve.

## Discussion

The RPF terminology generally indicates a clinicopathological entity with the characteristic of the presence of a sclerotic tissue in the periaortic and peri-iliac retroperitoneum, often enclosing surrounding structures such as the inferior vena cava and the ureters [5]. Idiopathic RPF belongs to the group of the disease having a connection with immunoglobulin G4 (IgG4), a disorder mediated by immunological reaction, affecting various organs (salivary glands, gallbladder, pancreas) [4].

When renal failure or obstructive uropathy occurs (42%-95%), only then the correct diagnosis is made. The most common presenting symptom is flank, dorsal, and/or abdomen pain. Other unusual symptoms are fever, abdominal angina, testicular pain, oedema, intermittent claudication, or macroscopic haematuria. Acute hypertension occurs in some patients with urinary tract obstruction, suggesting a possible role of an increase in renin levels [6]. In our case also, the patient presented with symptoms and signs suggestive of AKI with elevated blood urea and creatinine levels, due to urinary tract obstruction caused by fibrosis around the aorta and bilateral common iliac veins, which is the most common site for fibrosis.

Imaging modalities such as CT and MRI are the most reliable studies, hence are necessary for the diagnosis and treatment of RPF and can help to distinguish between idiopathic and secondary causes [7]. Ultrasound should be done as the first investigation of choice, specifically in the evaluation of a patient with renal failure. IRPF is seen as an isoechoic or hypoechoic mass on ultrasound, which can involve the ureters and, therefore, causes hydronephrosis involving one or both kidneys [7]. Similar findings have been noted in ultrasonography and CT scans of the abdomen in our patient.

To stop the fibro-inflammatory reaction from progressing further, the acute phase reaction, and its systemic manifestations, inhibit or improve the compression of the ureters or other retroperitoneal structures, for which corticosteroids are the mainstay of therapy. Tamoxifen or immunosuppressants such as azathioprine and cyclophosphamide is a preferable alternative when steroids are contraindicated, but with a higher relapse rate compared to steroids, thus used as second-line drugs in patients who are refractory to steroids. Biological agents, such as rituximab, tocilizumab, and infliximab, have been tried in refractory IRPF cases, but less data is available to date [4]. Surgery is generally done to relieve obstruction of the ureters; Open ureterolysis with intraperitoneal transposition and omental envelope of the ureters is opined as the best approach for surgical intervention. Surgical interventions do not help in preventing the progression of the disease or in its recurrence. Surgery must be given with systemic steroid therapy. The conservative management comprises systemic treatment with stent placement in ureters or nephrostomies has been suggested, therefore reserving surgical management in refractory cases [7]. Our patient was treated with a low-dose systemic steroid along with treatment for AKI, and bilateral ureteric stent placement was done by the urologist to relieve the ureteric obstruction.

## Conclusions

RPF is an uncommon disease that is difficult to diagnose, creating a therapeutic challenge for retroperitoneal diseases. The diagnostic modalities of choice are CT and MRI, showing the characteristic fibrosis surrounding the aorta and ureter. Medical management with glucocorticoids is the backbone of treatment, whereas tamoxifen, biological agents and immunosuppressants are tried in case of incomplete response or contraindication to steroids. Surgical management has been reserved for refractory cases, mainly for severe ureteric obstruction; conservative treatment with systemic therapy is the standard treatment.
